# Mr. Blair's Reply to Dr. Beddoes

**Published:** 1801-06

**Authors:** W. Blair

**Affiliations:** Great Russell Street


### *To the Editors of the Medical and Phyſical Journal.*

Gentlemen,

Although the honeſt oppoſition which has been made to the uſe of nitrous acid as a ſubſtitute for mercury in the venereal diſeaſe, is ſaid to have ariſen from prejudice or avarice, and to have been continued by malicious cabals, or foul practices, tices,* I ftill venture to exprefs my conviction, that the latter remedy will never fupplant the former. There is even caufe to fufpect that the confident affertions of fome Britifh practitioners on this fubject, have diminifhed their credit in other refpects among our medical brethren upon the continent; this appears from different publications that have fallen into my hands, and from none more clearly than a paffage in a recent work of Dr. Aubert on the Cow-pox.†

A correfpondent of yours, who boafts that he has arrived, "if enquirers ever did arrive," at inconteftible certainty, feems neverthelefs willing to adopt a method of afcertaining the anti-venereal virtues of the acid, which muft bring this controverfy "at once to a decifive iffue." I beg leave therefore, to affure you of my readinefs to concur with any number of refpectable practitioners in the execution of fo defirable a plan: It is my wifh, however, to ftand forward rather as an obferver than a coadjutor, to mark what can be effected by others, rather than to interfere with their meafures. The part I have already taken in this inveftigation,‡ forbids me to hope for complete fuccefs in any future attempt; but it becomes me not to fhrink from any further degree of trouble which my opponents deem requifite to afcertain the truth. I am, &c.

*Great Ruffell Street*

*May* 23, 1801.

W. BLAIR.

* See Dr. Beddoes's Communication. London, 1800, 8vo. at pages 5, 9, 43, 52, &c.

† "*Pouvons-nous croire fans réferve ce que les Anglais affirment?* Les médecins de cette nation font, à jufte titre, eftimés dans tous les pays; mais cet ordre refpectable renferme quelques novateurs qu'on fufpecte, et dont on fe défie avec raifon. Ce font ces derniers qui ont fait dire aux Allemands, que les Anglais font empiriques, et au Français, qu'ils annoncent fouvent des fait qui n'ont aucune réalite; on pourrait citer entrautres les cures nombreufes attribuées aux acides nitrique, muriatique, &c. que l'experience a démenties a Paris; ou l'on employa ces nouveaux remèdes contre le venin fyphilitique, pendant long-tems, avec laplus fcrupuleufe exactitude, et toujours fans fuccès, on du moins, fans que ces médicamens détruififfent les effets du virus fyphilitique auffi complétement et d'une manière auffi fure, qu'on l'avait annoncé. On pourrait rappeler encore les merveilles de la médicine pneumato-chimique, fi vantée par BEDDOES, et rejetée à cette heure, par l'auteur même qui l'avoit prônée."

*Rapport fur le Cow-pox, par* A. AUBERT, *D. M. Difcours Préliminaire, p. vii.*

‡ Effays on the Venereal Difeafe, Part I. and II. Symonds, London, 1799 and 1800.

